# Speciation of vanadium and the interacted solid surface of δ-alumina adsorbent in aqueous media in presence of humic acid

**DOI:** 10.1038/s41598-024-62941-z

**Published:** 2024-06-06

**Authors:** Ashraf A. El-sayed

**Affiliations:** Analytical Chemistry Department, Hot Labs Center, Egyptian Atomic Energy Authority, Cairo, 13759 Egypt

**Keywords:** Biogeochemistry, Environmental sciences

## Abstract

Speciation of vanadium elements in the presence of δ-alumina in aqueous media was studied to simulate the environmental impact of soil/sediment–water interacted system. Factors affecting this process are pH, presence of humic acid, and δ-alumina concentrations as an abundant sediment/soil components. Different species of both vanadium and surface of δ-alumina were deduced theoretically using MintaqA2 programme. Due to the effect of pH, the anionic species of vanadium at pH 1–3 is prevailed and changed to cationic species at pH range 6–10 at different levels of alumina. Additionally, based on the effect of alumina concertation, high percent uptake, almost 100% was found at 10.0 g/1 concentration of alumina while at level of 0.2 g/1 alumina, the maximum adsorption of vanadium was become 91%. The effect of humic acid on the speciation behavior of vanadium (V) was also studied and compared with that of vanadium (IV) based on XANES (X-ray absorption near edge structure). Adsorption behaviors were studied at concentration 4.71E-4M for vanadium at 0.1M ionic strength. The mechanism of vanadium adsorption in the presence of alumina under the same working conditions was studied and explained based on TLM (Triple layer model) where the results proved good validation and verification of the practically produced data.

## Introduction

The interesting in studying the behavior of vanadium is coming mostly from its increased dispersion in the biogeosphere and the increased risk of its toxic effects^1^. Therefore, the needs to follow up its removal and determination not only in the water but also in sediment are very important^2,3^ because it can be introduced into the water ecosystem in both dissolved and/or adsorbed on solid particulate. Under different values of pHs, vanadium can be mobilized to the aqueous phase with varieties of several species. Investigation with K-edge XANES spectroscopy have shown that most oxic soils contains a mixture of vanadium(IV) that has stereo structure of octahedrally coordinated complex and surface-bound vanadate(V) specially on aluminium (hydr)oxides, while acid organic soils form the organically complexed vanadyl (IV)^4,5^. In reduced environments, as in sediments, it was suggested that the vanadium consists of a mixture of organically complexed V (IV) and V(III) species. Where, according to published works, the species of V (III) hydrolyses to V(OH)^2+^ at low pH, at very low ionic strength, and hydrolysed further to V(OH)_2_^+^ at pH 4.7. While, V(IV) occurs in solution as the oxovanadium (IV) or vanadyl (IV), VO^2+^, which is characterized by its strong V=O double bond, with a bond length of 1.588 Å. Organic surface horizons in forest soils and surface waters with low pH values are examples of environments where vanadyl (IV) is probably the most stable vanadium species^6,7^. The greatest source of vanadium is from combustion of petroleum, coal and heavy oils beside its release to soil and water from weathering of rocks, rather than anthropogenic sources. Previous works reported and proved that different ways^8–10^ leading to vanadium leaching were potentially expected from mixed coal and petroleum coke combustion fly ash. Additionally, the lower vanadium oxidation states can be found in gasification systems which are generally nonvolatile^11^. Generally, the different valences of vanadium are normally occurring in several oxidation states in nature which are; V(III), V(IV) and V(V)^12–14^. That can be explained based on the ecological system, for instance, V(III) is mostly formed under acidic and reduced conditions of soil environments; V(IV) is prevalent in slightly acidic and moderately reduced condition of groundwater and soils which is exposed to industrial activities and acid rain and V(V) occurs in a normal condition of oxic waters at a wide range of pH^15,16^. Furthermore, speciation of vanadium can be affected by its concentrations, where, the monomeric pentavalent forms, such as H_2_VO_4_^−^ and HVO_4_^2^, are dominant in dilute solutions and very common in soils ([V]_*T*_ = 10^−5^ to 10^−6^ M. On the other hand, the oligomeric pentavalent forms, such as V_4_O_12_^4−^ and V_10_O_28_^6−^, are most predominant species in concentrated solutions of vanadium^17,18^. Therefore, the release of metals to the aquatic environment is definitely controlled by several factors such as; pH, ionic strength, organic ligands and the presence of different type of sorption particles which cause changing in physico-chemical conditions. That mean sorption of several cations and/or anions at solid–liquid interface may significantly alter the types of species and consequently the mobility of the ions. In this concern, many adsorbed particles in natural system are well-known and characterized by a great diversity of structures such as, rock minerals, clays, metal oxides (alumina), organic particles, humus macromolecules and inorganic particles coated with organic matter. Naturally, the amount of alumina predominates as alumina silicate, and present in several other geological formations and present in different environmental compartments of terrestrial origin that represents 1.8% by weight and exist in the earth's crust with a mean abundance of at least 1.0 ppm. Also, nowadays Al_2_O_3_ nanoparticles are used in various adsorbent and catalyst applications, including catalyst adsorption in polyethylene production, hydrogen peroxide synthesis, and as a selective adsorbent to remove different chemicals from gas streams. Aluminum oxides are used in varieties of application such as; ceramics, refractories, and abrasives because of their hardness, chemical inertness, high melting point, non-volatility, and resistance to oxidation and corrosion. Also, humic acid as an organic material predominate in the environment, occurring among the hotter decomposition products in upper surface parts of soil and has a potential reductive ability and plays a role in decreasing the toxicity of various oxyanions of some heavy metals^19^. The aim of this work is to use alumina in a trial to explicit its role in removal of toxic species by physico-chemical processes that could be occur in the bulk of solution or at the phase interface. The adsorption behavior of vanadate species on the surface of alumina was tested as a function of pH and adsorbent concentration. The presence of natural organic materials such as humic acid was also studied.

## Results and discussion

### Characterization of δ-alumina

**Figure 1:**
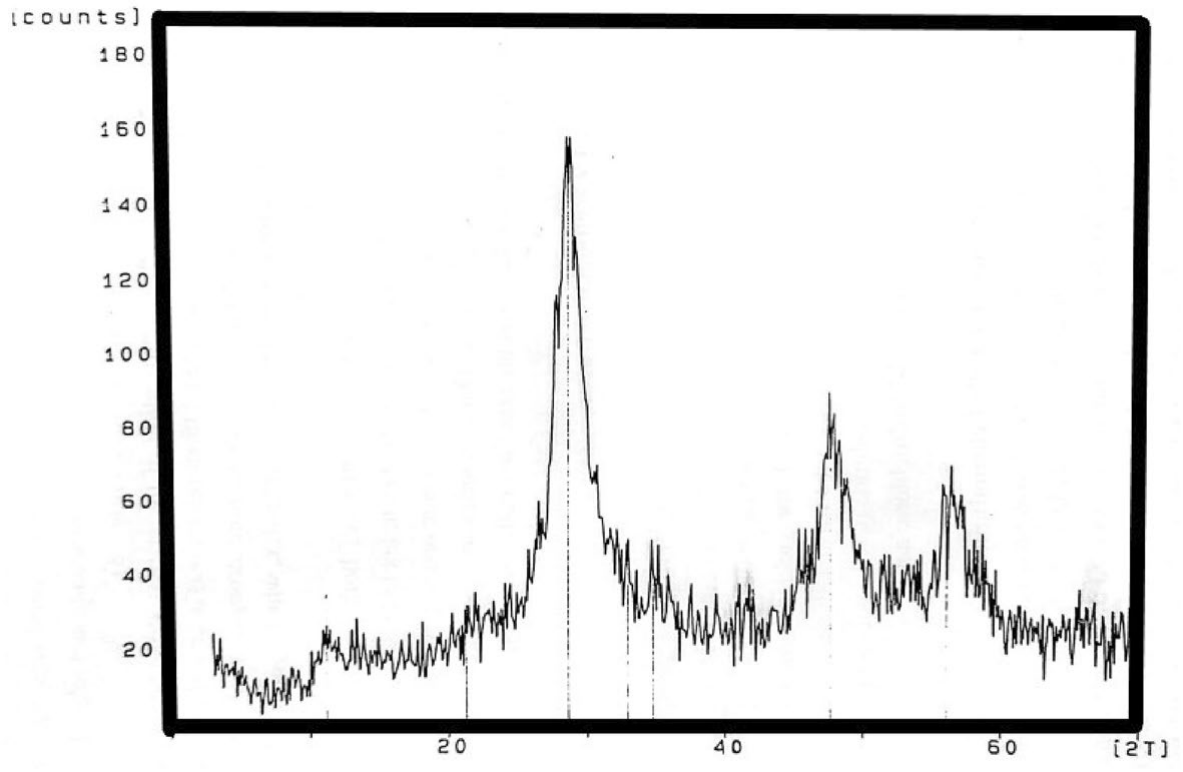
The XRD diagram of δ-alumina.

Based on the X-ray diffraction and the other work published on the δ-alumina, it was deduced that it is amorphous in shape with surface area of 130 m^2^/g with mesh size less than 0.2 mesh. From x-ray diffraction patter, Fig. 1 it is shown that the peaks are wide which support the amorphous characteristics. Also, δ-alumina was calcinated at 650 and it is one of metastable transitional forms of boehemite amorphous Al_2_O_3_ to → γ-Al_2_O_3_ → δ-Al_2_O_3_ → θ-Al_2_O_3_ → α-Al_2_O_3_^20^ which is in the chemical form Al_2_O_3_. In this work, δ-alumina was used based on its easily attacked by hydration and protonation process which is the keyword of the adsorption process. Delta alumina (δ-Al_2_O_3_) has a tetragonal superstructure of the spinel lattice with a triple cell of γ-Al_2_O_3_.

### Alumina surface speciation

**Figure 2:**
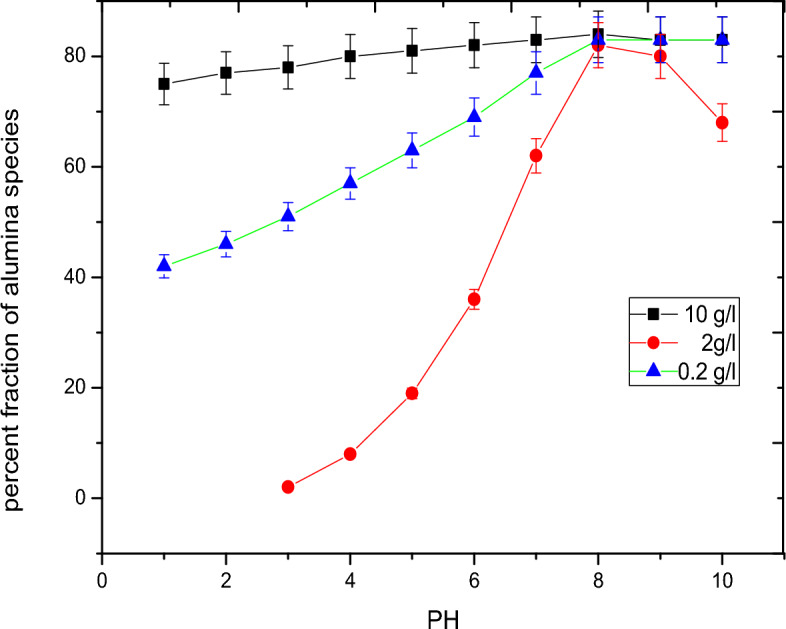
Surface speciation diagram of alumina for AlOH.

**Figure 3:**
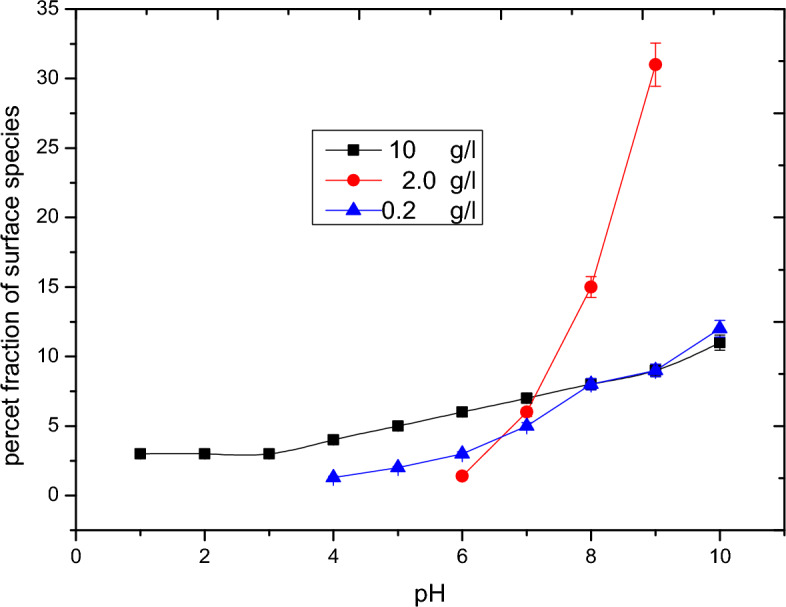
Surface speciation diagram of alumina for AlO^−^.

**Figure 4:**
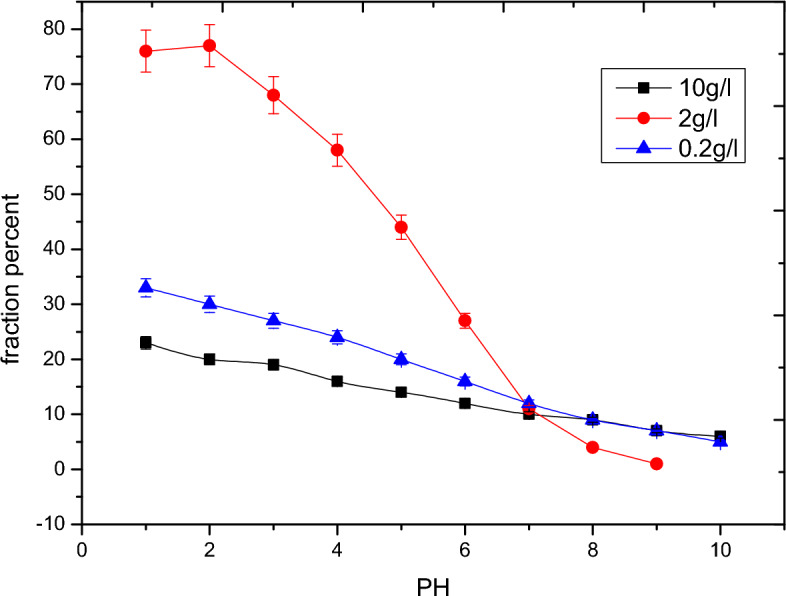
Surface speciation diagram of alumina of AlOH_2_^+^

Based on the triple layer model (TLM) the species of alumina in a aqueous system can be categorized into three main species; AlOH, AlO^−^ and AlOH_2_^+^. Figures 2, 3 and 4 outline the effect of pH on the different species of alumina. These species show how formation of the complex compounds of the adsorbent with the adsorbate molecules based on the effect of pH.

For all experiment an amorphous alumina was selected to the whole work and its structure was explained based on the presence of active sites that lead to the adsorption of metal through making of double and/or triple layer (TLM/DLM) at the phase interface either by inner or outer sphere complex formation. All vanadium forms are strongly bound to the sorbents where V(V) is bound as a bidentate complex on aluminum (hydr)oxides, and with a stronger affinity than that of other anion species such as, orthophosphate. V(IV) is strongly complexed to natural organic matter specially in the anoxic circumstances. The suggested chemical reactions mechanism when a solid oxide or hydroxide is brought into contact with an aqueous electrolyte solution can be described based on chemical reactions mechanisms as show in the following equations, where, the notation ISOH(s) represents a single protonated oxide site in an oxide or hydroxide solution. Also M and L represent metal cations, metal anions and/or ligand.

Surface reactions:

1. Proteolysis
  $$\begin{gathered} {\text{ISO}}^{ - } \left( {\text{s}} \right) + {\text{H}}^{ + } \left( {{\text{aq}}} \right) = {\text{ISOH}}\left( {\text{s}} \right) \hfill \\ {\text{lSOH}}\left( {\text{s}} \right) + {\text{H}}^{ + } \left( {{\text{aq}}} \right) = {\text{ISOH}}_{{2}}^{ + } \left( {\text{s}} \right) \hfill \\ \end{gathered}$$
2. Exchange reaction
  $$\begin{aligned} & {\text{ISOH}}\left( {\text{s}} \right) + {\text{pM}}^{{{\text{m}} + }} = \left( {{\text{ISO}}} \right){\text{aM p}} + {\text{aH}}^{ + } {\text{aq}} \\ & {\text{ISOH}}\left( {\text{s}} \right) + {\text{pM}}^{{{\text{m}} + }} {\text{aq}} + {\text{qLl}} - \left( {{\text{aq}}} \right) = \left( {{\text{ISO}}} \right)_{{\text{a}}} {\text{MpL q}}\left( {\text{s}} \right){\text{ p}} + {\text{aH}}^{ + } \left( {{\text{aq}}} \right) \\ & {\text{ISOH}}\left( {\text{s}} \right) + {\text{pM}}^{{{\text{m}} + }} {\text{aq }} + {\text{H}}_{{2}} {\text{O}}\left( {\text{ I}} \right) = {\text{ISOMOH}}\left( {{\text{m}} - {2}} \right)\left( {\text{s}} \right) + {\text{2H}}^{ + } \left( {{\text{aq}}} \right) \\ & {\text{ISO}}^{ - } \left( {\text{s}} \right) + {\text{H}}^{ + } \left( {{\text{aq}}} \right) = {\text{ISOH}}\left( {\text{s}} \right){\text{ lSOH}}\left( {\text{s}} \right) + {\text{H}} + \left( {{\text{aq}}} \right) = {\text{I}} - {\text{SOH}}/\left( {\text{s}} \right) \\ \end{aligned}$$
3. Overall Reaction
  $${\text{aISOH}}_{{({\text{s}})}} + {\text{pM}}^{{{\text{m}} + }}_{{({\text{aq}})}} + {\text{qL}}^{{{\text{l}} - }} ({\text{aq}}) + {\text{nH}}^{ + }_{{({\text{aq}})}} + {\text{rOH}}^{ - }_{{({\text{aq}})}} = ({\text{ISO}}){\text{aM}}_{{\text{p}}} ({\text{OH}}){\text{rHn}}\;{\text{L}}^{\updelta }_{{{\text{q}}({\text{s}})}} + {\text{aH}}^{ + }_{{({\text{aq}})}}$$

This oxide surface can act as a weak acids or bases in solution which undergoing protonation in response to change in solution pH. Additionally, the surface can enter into complexation reaction with other ions in solution, by which protons are released to solution when an uncomplexed metal has been adsorbed. This type of alteration in surface charge resulting from chemical reactions is frequently referred as specific adsorption.The triple layer model (TLM) is a surface complexation model of the oxide-aqueous solution interface developed from the conceptualization of electrical double layer discussion. Generally, reviews and representative application of this model have been given elsewhere^21^. Like other model TLM employs assumptions concerning the nature of surface complexes and the conditional equilibrium constants that describe their formation. TLM also postulates that the surface species, except SOH, SOH^+^(s) and SO^−^, are outer-sphere complex. Since the triple layer model permits the formation of outer-sphere surface complexes it can be applied to describe the interaction between surface hydroxyl and a solution containing a univalent background electrolyte, such as sodium perchlorate. The reactions of complex formation that have been considered in the model include the previous equations and the conditional equilibrium constants defined in that model as K_a1_, K_a2_ and a, b, as shown in the subsequent equations. Based on the above theoretical considerations, the adsorption behavior of vanadate (VO_3_^−^) on the surface of alumina was examined as a function of pH, adsorbent concentrations, practically and theoretically. Effect of naturally organic material, humic acid was also studied. Therefore, the adsorption behaviors of this element were studied in details to monitor, control as well as speciate its presence in the environment.

$$\begin{aligned} & {\text{Ksa1}} = \left[ {{\text{ISOH}}} \right]\left[ {{\text{H}}^{ + } } \right]/\left[ {{\text{ISO}}^{{{2} + }} } \right] \\ & {\text{Ksa2}} = \left[ {{\text{1S}}0^{ - } } \right)\left[ {{\text{H}}^{ + } } \right]/ \left[ {{\text{ISOH}}} \right]. \\ & {\text{Asa}} = \left[ {\left( {{\text{ISO}}} \right){\text{aMpL q}}\left( {\text{s}} \right){\text{ p}}} \right]\left[ {{\text{aH}}^{ + } } \right]/\left[ {{\text{aISOH}}\left( {\text{s}} \right)} \right]\left[ {{\text{pM}}^{ + } } \right]\left[ {{\text{qLr}}} \right] \\ & {\text{K}}_{{{\text{ft}}}} = \left[ {{\text{ISOMOH}}\left( {{\text{m}}^{{ - {2}}} } \right)} \right]\left[ {{\text{ 2H}}^{ + } } \right]/\left[ {\text{ ISOl l}} \right]\left[ {{\text{M}}^{{{\text{m}} + ^{\prime}}} \, } \right]\left[ {{\text{H}}_{{2}} {\text{O}}} \right] \\ & {\text{k}}_{{{\text{asb}}}} = \left. {\left( {{\text{ISO}}} \right){\text{aHnL}}\left( {\text{s}} \right)} \right]\left[ {{\text{aH}}^{ + } } \right]/\left. {{\text{alSOH}}\left( {\text{s}} \right)} \right]\left[ {{\text{ qL}}^{ - } } \right]\left. {{\text{nH}}^{ + } } \right] \\ \end{aligned}$$

Therefore, the mechanism of the adsorption process can be explained based on the solid surface of alumina where firstly, it can chemisorbs a monolayer of water when exposed to moisture where in dried state the outermost layer of Al_2_O_3_ surface contains only oxide ions regularly arranged over aluminum ions in octahedral sites in the lower layer. Additionally, hydration water is chemisorbed and the top layer of oxide ions is converted to hydroxyl ions. Hydroxyl ions coordinated with aluminum cations constitute the reactive sites (Al–OH) on alumina surface. In this concern, more than one type of surface OH group is discriminated based on stereo chemical composition. The number and type of surface OH groups depend on the preferentially exposed surface plane together with the distribution of aluminum ions. Other mechanism was reported by Hiemstra's model of aluminum oxide surface^22^ which considers only two active sites, singly and doubly coordinated Al atoms on surface. Since the coordination of both oxygen atoms and hydroxyl groups on a partially dehydroxylated alumina surface differs from each other by the number and the type of surrounding metal atoms, five types of active sites were differentiated on the basis of potentiometric titration.

### Effect of alumina concentrations and pH

**Figure 5:**
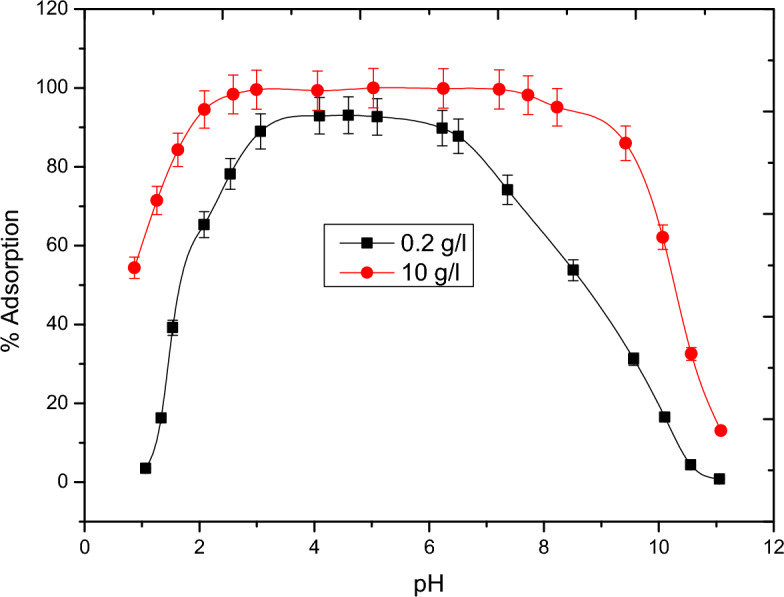
The effect of pH and alumina concentration on the adsorption of vanadium.

**Figure 6:**
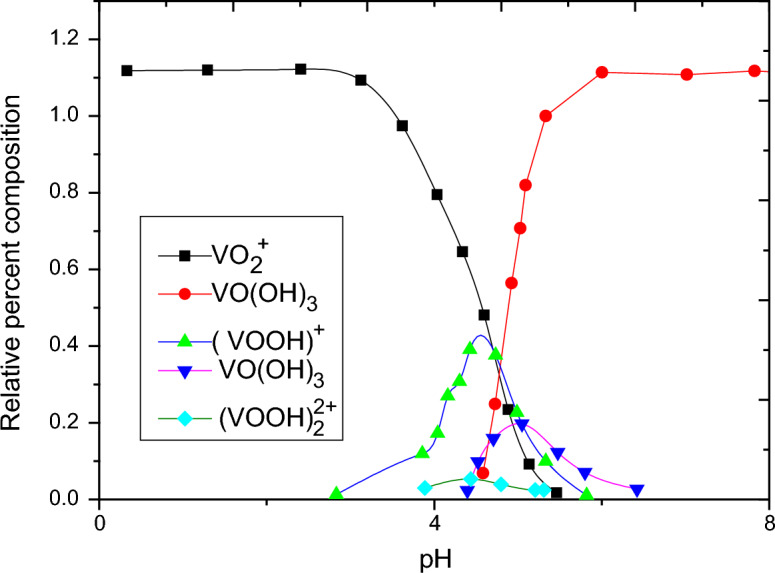
Speciation diagram in aqueous media as the effect of pH.

**Figure 7:**
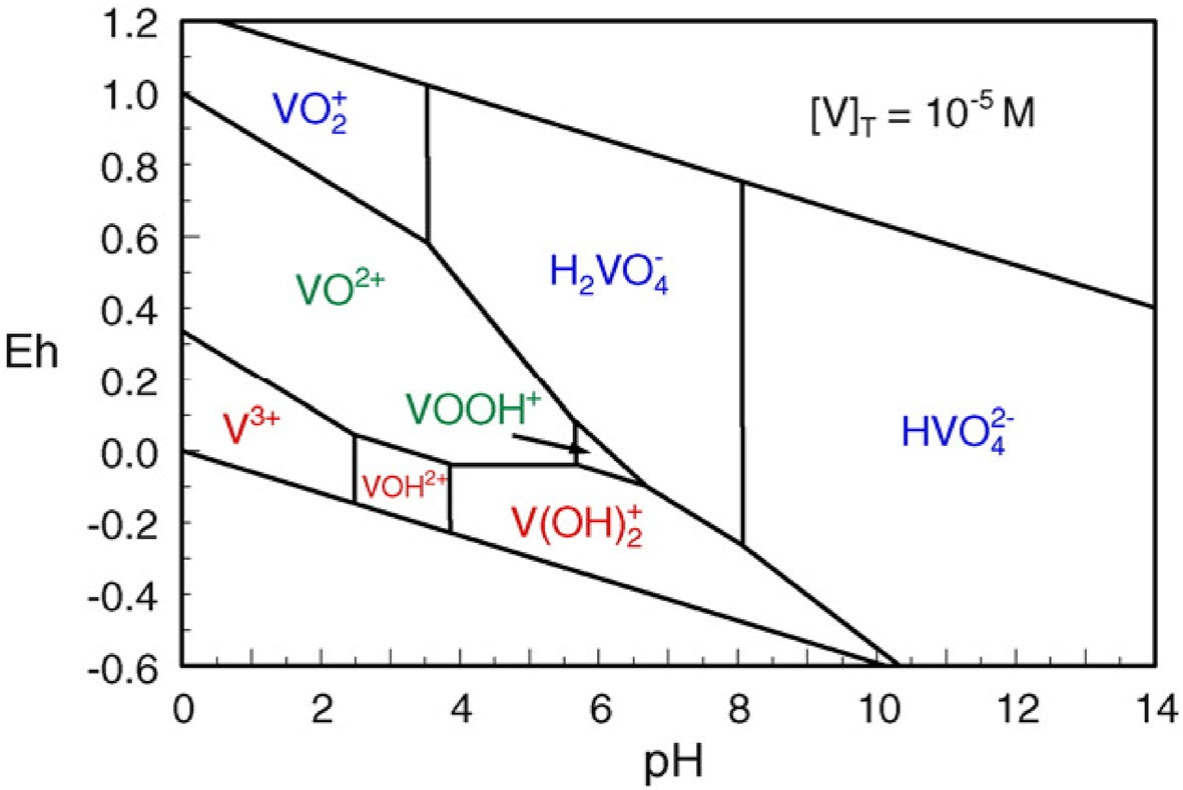
Effect of pH on the electrolyte potential of the formation of differet species of vanadium in a aqueous solution.

Figure 5 shows the adsorption behavior of vanadate on both 10 g/1 and 2 g/1 of alumina. It was found that the adsorption increase from zero to nearly 100% within a narrow range of 1–3 pH unit contrary to normal other oxyanion species as chromate^22^ which increases by increasing pH till become constant t. At the same time, increasing pH show decrease in the adsorption of metavanadate. This behavior can be explained based on the formation of different species according to the speciation diagram of vanadium, Fig. 6. Also, the common electrolyte effect on the speciation of vanadium in solution is shown in Fig. 7. the In presence of alumina, it was found, theoretically, that at low pH, positive and neutral species H_2_V_2_O_4_^+^, HVO_2_^2+^, H_2_V0_3_^+^ and H_3_VO_4_ are predominant which can be sorbed on the charged site of hydrolyze silanol group of AlO^­^ symbolized as (ISO) While at higher pH the formation of negative species, which are sorbed on the positive silanol group AlOH^2+^. The species of vanadium are VO_3_^−^ and VO_3_(OH)^−^. These behaviors were found for 10 g/1 and 0.2 g/1 of alumina due to pH effect in the aqueous phase. Based on the above theoretical consideration the equatios of adsorption behavior^23^ can be represented as follows:

$$\begin{aligned} & {\text{AlOH}} + {\text{VO}}_{{3}}^{ - } = {\text{I SOH}}^{ + } {\text{VO}}_{{3}}^{ - } \quad {\text{at lower pH}} \\ & {\text{AlO}}^{ - }_{{\text{S}}} + {\text{H}}_{2} {\text{VO}}_{3}^{ + } = {\text{AIO}} - {\text{H}}_{2} {\text{VO}}_{3}^{ + } \quad {\text{at higher pH}} \\ \end{aligned}$$

### Theoretical simulation of adsorption behavior

**Figure 8:**
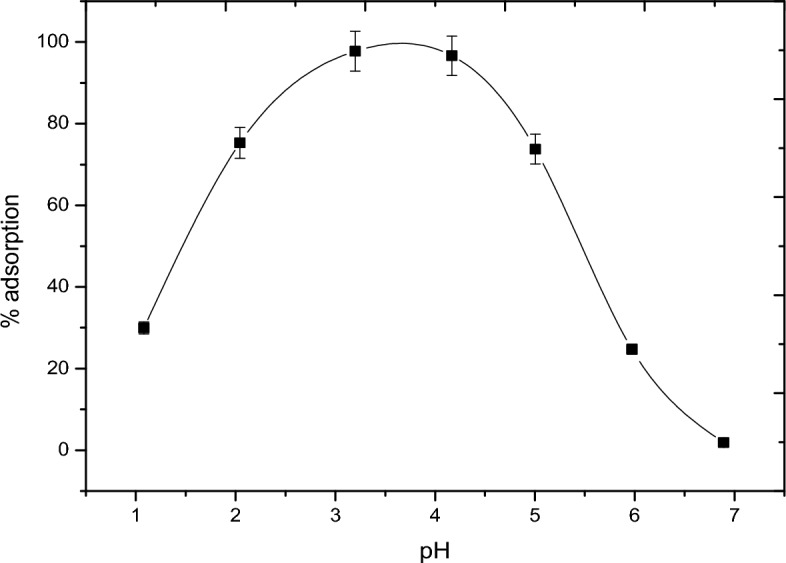
Theoretical adsorption behavior of vanadium (V) on alumina using MinteqA2 programme.

Simulation of adsorption behavior of vanadium theoretically was elaborated using MinteqA2 software. This theoretical study was done based on the TLM suggestion in which three hypothetical planes were constructed according to the formation of the inner and outer Helmoholtez planes. For oxide surface, the ions H^+^ and OH^-^ are also assigned to inner most planes and beyond this surface plane a layer of ions attracted to surface by specific chemical interaction is formed. The location of the center of these ions is known as the inner Helmholtz plane. The species usually assigned to this plane include chemically adsorbed metal and legends as well as weakly adsorbed electrolyte ions. Figure 8. Represent the theoretical diagram of the adsorbed vanadium oxy anion species forming the inner sphere complex with the charged surface of alumina through formation of covalent bond. The process of adsorption takes places based variable charged species that ca be formed by the effect of pH. For example, the oxyanion VO_3_^−^, the conjugate bases of monoprotic acids was formed which occurs at/or near the pKa of acids. Therefore, the mechanism or adsorption of oxyanions of vanadium involves the replacement of surface –OH and/or –OH_2_ on variable charge surface.

### Effect of humic substances

**Figure 9:**
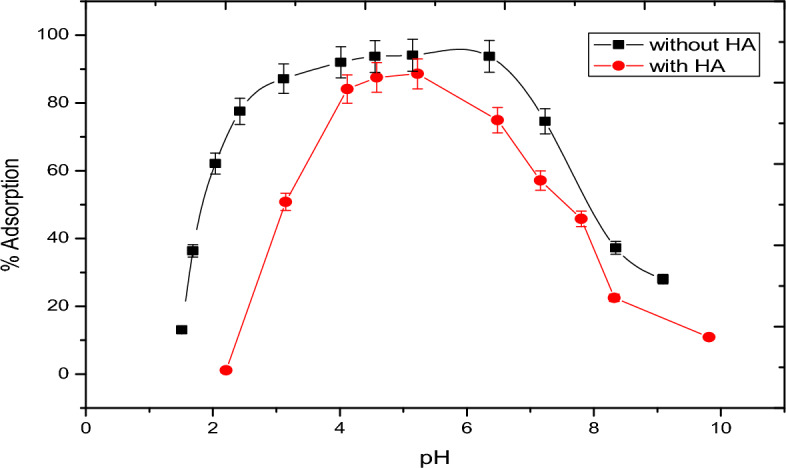
Adsortion behavior of vanadate in presence and in absence of humic Acid materials.

**Figure 10:**
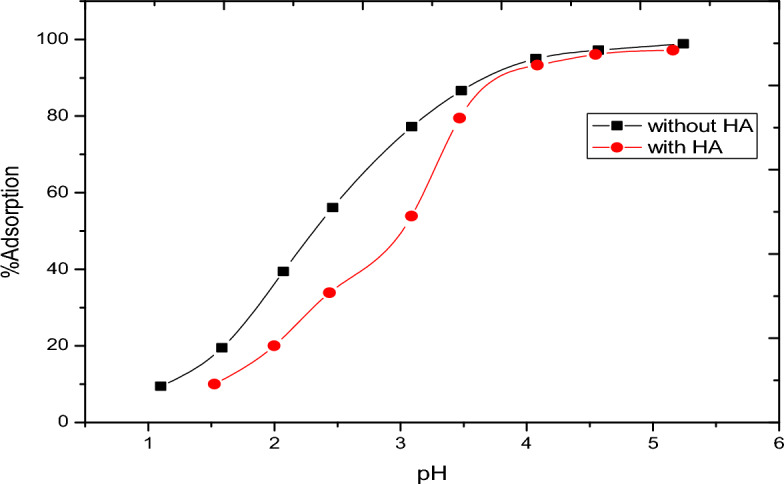
Adsortion behavior of vanadium (4+) in presence and in absence of humic Acid materials.

**Figure 11:**
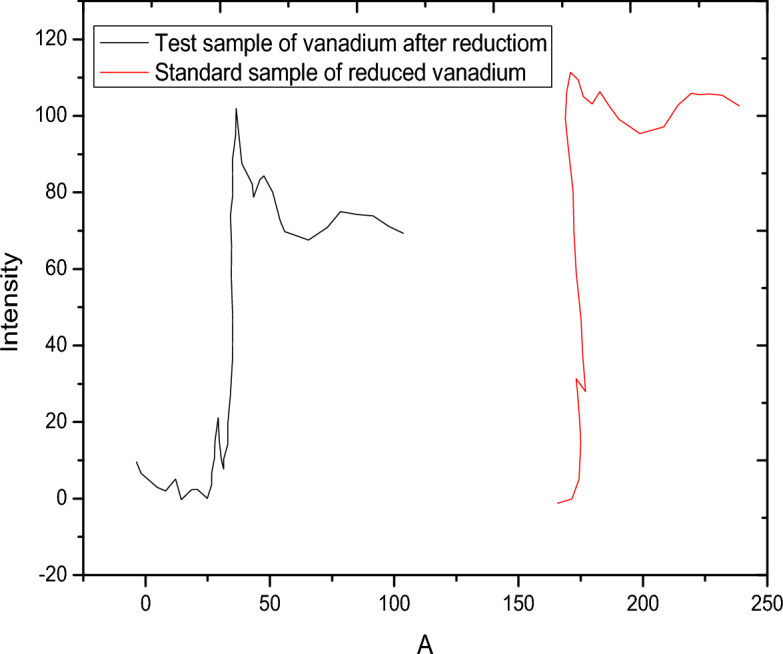
Comparison between vanadyl ion on alumina/humic subsataces after adsorption process of the metavanadate.

The presence of organic rich materials was relevant in the nature^24^. The role of solid-water interfaces and surface controlled reaction is a prerequisite for understanding the retention of natural organic matter and the stability/transport of both organic matter and inorganic colloids the subsurface soil environments. In this case, pH-adsorption studies of vanadate and vanadyl will be performed in the presence of humic acid. Figures 9 and 10 show the fractional adsorption of vanadate as NH_4_VO_3_ and vanadyl as VOSO_4_ as a function of pH. In Fig. 9, adsorption behavior of vanadate in presence of alumina with and without humic acid is the ·same; increasing adsorption with increasing pH. However, adsorption in case of humic acid is lower, starting at pH 2 with nearly 2% adsorption higher than that in absence of humic acid starting at pH 1 with l0% adsorption. This could be explained based on the formation of VO_2_ vandyl species according to one electron reduction mechanism^25^. At the same time, the adsorption of vanadyl with and without humic acid, Fig. 10 was also studied. This behavior showed that the humic acid slightly affect the adsorption of vanadyl species. Figure 11 shows a comparison between the vanadium in presence of humic substance with standard vanadyl compound using XANS, the figure indicates the adsorption of vanadate is accompanied with the reduction process to vanadyl ion. It is worth to mention that the metal binding capacity of humic substance find its origin in presence of functional groups. Due to the large variety of different functional groups humic substance should be considered as heterogeneous ligand^26–30^.

### Significance of the adsorption process

Vanadium is considered important metal with wide uses in different industries due to its unique chemical and physical properties. Therefore, the recovery of vanadium (V) is obligatory because of the lack of its raw materials. Several methods can be used to restore vanadium (V) from aqueous solutions. Where it is used to produce steel with rust-resistant elements and as a carbide stabilizer in the manufacture of steel, especially for nuclear applications. Also, vanadium pentoxide is used as a catalyst, as a mordant for dyeing and printing fabrics, in the manufacture of aniline black, and in the ceramics industry. Other vanadium compound such as vanadium-gallium is used to produce superconducting magnet. This study develops an effective process for the recovery of vanadium (V) by using the adsorption method. In this direction, the percent adsorption and the capacity is compared to other work as shown in the following in Table 1. It was found that the delta alumina is very efficient as significant materials for removal of meta vanadate anions.

**Table 1:** The performance of alumina adsorbent comparing to other new prepared.

Adsorbent	Refs.	(mg/g)	(% removal)
AlO-1	^31^	0.35	75
AlO-1FHY	^31^	0.45	90.5
AlO-2FHY	^31^	0.47	98.5
MT-BElFEHY1	^31^	0.53	98.2
Alumina	This work	0.61	95

## Experimental

### Material and methods

All reagents are analytical grade and used without further purification. Amorphous alumina was purchased from Degussa, Germay. Humic acid was of natural origin ad imported by Aldrich Co., England, and used without any further treatment. Experiments were performed in tightly capped Teflon container under nitrogen atmosphere at 25 ± 0.1 C for the preparation of alumina suspension which was freshly prepared by using weighed amounts 0.2 and 10 g/1 of alumina in 100 ml measuring flask followed by adding the adjusted concentration of aqueous solution of vanadium. The ionic strength of the aqueous medium adjusted to 0.1 M by sodium perchlorate and the suspension of alumina was then homogenized for one hour in an ultrasonic water bath. For three stock suspensions containing different amounts of alumina, the concentration of vanadium was kept constant and series of 10 experiments were carried out at different pH values from 1.0 to 10.0. In an ascending sequence for each pH, 15.0 ± 0.1 ml of the homogenized suspension were transformed to quick sealed tube, purged with nitrogen then shaken for one weak in dark. After the shaking period, the 10 quick sealed tubes were centrifuged for 60 min at 20,000 rpm at room temperature, using Beckman-Laboratory ultracentrifuge, Germany. Two milliliters supernatant aqueous medium were then removed and submitted for analysis. Adsorption behavior and speciation were simulated using geochemical model, MinteqA2, and chemical equilibrium model. The percent sorption ad maximum capacity were calculated based on the following Eqs. (1) and (2):

1 $$\% {\text{ads}} = = \left( {{\text{Co}} - {\text{Ct}}} \right)/{\text{Co}}$$

2 $${\text{Q}}_{{\text{t}}} = \left( {{\text{C}}0 - {\text{Ct}}} \right){\text{V}}/{\text{m}}$$

Co (mg/L): initial vanadate concentration; Ct (mg/L): the concentration corresponding to a reaction time of (t) in solution; m (mg): the mass of the sorbent; V (L): the volume of solution.

### Instrumentation

The Inductively Coupled Plasma (lCP-AES) was accompanied with a compact tuned-oscillator and high-resolution Echelle-grating single pass prism to cover wavelength range 190–800. Perkin Elmer, USA. The samples were introducing by peristaltic pump and a Hildebrand Grid nebulizer. The system includes a plasma spec Leeman, 2.3 KW generators with 3-turn copper load coil. An analytical program was established for both calibration and routine analysis for vanadium element. The selected analytical wavelengths represent the characteristic lines. The degree of crystallinity of alumina was tested using Phillips PW 1140 X-ray,USA. generator in combination with vertical goniomter and proportional counter using copper X-ray tube operating at a power level of 1400 W.

### Ethical approval

This article does not contain any studies with human participants or animals performed by the authors.

### Consent to participate

The author state that no irreconcilable situation with any one cooperate with the author.

## Conclusion

Quantitative adsorption of vanadium from aqueous solution on to alumina can be achieved using 0.2 g/1. The presence of naturally humic substance may affect both the adsorption and speciation of the oxyanions species in heterogeneous system. Generally, alumina was found to have potential higher capacity for oxyanions species.

## Data Availability

All data generated or analyzed during this study are included in this published article.
